# The Methylation of the *CYP11A1* Gene Affects the Expression Pattern in Different-Diameter Granulosa Cells of Qira Black Sheep

**DOI:** 10.3390/genes16121426

**Published:** 2025-11-29

**Authors:** Chunjie Liu, Peilin Guo, Linlin Pei, Wenhao Wang, Andi Qiao, Xin Xu

**Affiliations:** 1 Key Laboratory of Livestock and Forage Resources Utilization Around Tarim and Key Laboratory of Tarim Animal Husbandry Science and Technology, College of Animal Science and Technology, Tarim University, Alar 843300, China; 2 State Key Laboratory Incubation Base for Conservation and Utilization of Bio-Resource in Tarim Basin, College of Life Science and Technology, Tarim University, Alar 843300, China

**Keywords:** Qira black sheep, molecular-assisted markers, *CYP11A1* promoter, methylation, granulosa cells

## Abstract

Background: CYP11A1 (a key enzyme in the process of progesterone synthesis) expression is a crucial factor that promotes the proliferation and progressive differentiation of granulosa cells (GCs) to support oocyte maturation and ovulation in livestock. Changes in its expression may be related to DNA methylation, but the specific regulatory mechanism of this remains unclear. Methods: The qRT-PCR, western-blot, CCK-8 assay, RNAi, dual-luciferase activity assay and sulfite sequening were used to explore the effect of the activity and methylation level of the *CYP11A* promoter on the proliferation of GCs. Results: Our studies first identified that the activity of *CYP11A1* promoted the core genes that affected the superior growth of follicles in Qira Black sheep, and activity changes were related to the degree of DNA methylation. We further revealed that the DNA methylation of GCs at different developmental stages was mainly regulated by the promoter region, and also clearly defined the optimal window period for *CYP11A1* to affect the proliferation of GCs. Conclusions: In summary, our data reveals the epigenetic regulatory mechanism of GCs in follicle development provides a new perspective for understanding the molecular basis of reproductive cell differentiation, and offers important theoretical support for future research and basic application in the field of livestock reproduction. Discussion: This not only helps to improve the efficient breeding and production system of sheep and the process of genetic improvement, but also provides a theoretical basis for fundamentally accelerating the expansion, breeding, and genetic quality innovation of superior sheep breeds in China, and also offers accurate targets for sheep genetic breeding.

## 1. Introduction

As an important local sheep breed resource in Xinjiang, the genetic selection and breeding of Qira Black Sheep have progressed relatively slowly over a long period. Currently, molecular-assisted marker technology is regarded as a feasible strategy for achieving rapid selection and efficient breeding of this breed [1]. Numerous studies have indicated that the polymorphism and expression levels of the *CYP11A1* gene are closely associated with reproductive traits in sheep, and it is considered a potential molecular marker for improving litter size [2]. This gene encodes cytochrome P450 family 11 subfamily A member 1, a key enzyme in steroid hormone synthesis [3]. Located in the inner mitochondrial membrane, it catalyzes the conversion of cholesterol to pregnenolone—the initial step in steroid hormone biosynthesis [4,5]. Furthermore, CYP11A1 has been demonstrated to be involved in regulating various physiological processes in livestock, including reproduction, growth, metabolism, and stress response [6,7].

The crucial role of CYP11A1 has also been observed in other species. For instance, in pigs, its expression level changes dynamically during ovarian development and pregnancy, correlating closely with steroidogenic demands, suggesting its involvement in ovarian function regulation through gene–metabolite collaborative networks [8]. Simultaneously, retinoic acid can enhance ovarian steroidogenic capacity via the MESP2/STAR/CYP11A1 pathway, which is crucial for maintaining normal ovarian function and reproductive health [9]. Moreover, CYP11A1 expression exhibits stage-specificity during development; it is highly expressed in the early stages of follicular development in mice but decreases in mature follicles [10,11], indicating its potential core role in early steroidogenesis and follicular growth.

In livestock, bovine studies have further revealed the complex regulatory role of CYP11A1 in follicular physiology. In cattle, changes in the expression of this gene can serve as an indicator of the physiological status of follicles during growth and atresia; however, CYP11A1-mediated expression of steroidogenic genes is suppressed when follicular development becomes less dependent on FSH [12]. Additionally, *N*-carbamylglutamate and l-arginine may influence steroidogenesis in granulosa cells by modulating CYP11A1 expression [13]. On the other hand, environmental contaminants like PFOA can significantly inhibit steroidogenesis by downregulating CYP11A1 expression and may further impair mitochondrial function and cellular metabolism [14]. These findings suggest that the molecular marker effects of CYP11A1 may be partially mediated through glucocorticoids, yet its core regulatory mechanism in sheep remains to be fully elucidated.

As an important component of the local livestock industry, the reproductive performance, growth efficiency, and stress resistance of the Qira Black Sheep directly impact breeding profitability. Therefore, a systematic investigation into the function and regulatory mechanisms of the *CYP11A1* gene is of great significance for enhancing the productivity and adaptability of this breed. Beyond classical genetic regulation, epigenetic mechanisms such as DNA methylation and histone modifications play key roles in regulating CYP11A1 expression [15]. As observed in mammals, elevated DNA methylation levels can lead to down-regulation of CYP11A1 expression, consequently impairing steroid hormone synthesis [16]. Therefore, this study aims to investigate the impact of *CYP11A1* gene methylation on its expression and its role in follicular GCs of Qira Black Sheep. Exploring the stage-specific epigenetic regulatory mechanisms of *CYP11A1* is expected to provide new targets and strategies for genetic improvement of reproductive performance in sheep.

## 2. Materials and Methods

### 2.1. Ethical Statement

All experimental protocols involving *Ovis aries* strictly followed the relevant guidelines set by the Science and Technology Ethics Committee of Tarim University (Approval ID: TUEC2023-060).

### 2.2. GCs Obtain and Culture

Twelve healthy Qira Black sheep (aged 1.5~2 years, weighing 50 ± 2 kg) were purchased from the Cele County Conservation Farm in Hotan, Xinjiang, China, and were slaughtered at the local slaughterhouse. Twenty-four ovaries were obtained and transported to the laboratory in saline solution (0.9% NaCl) containing an antibiotic–antimycotic agent (1%) (100 IU/mL penicillin and 50 mg/mL streptomycin), maintained at a temperature of 30–35 °C. In the laboratory, ovaries were washed twice in warm physiological saline solution, and follicles were punctured with an 18-gauge needle attached to a 10 mL syringe to collect granulosa cells (GCs). The following three distinct groups of follicle sizes were used: Small (2~3.4 mm), Moderate (3.5~5.4 mm), and Large (>5.5 mm) in diameter, which correspond to the same groups used in a previous paper published [17].

Cells were cultured with DMEM/F-12 medium (Hyclone, Logan, UT, USA) supplemented with 10% fetal calf serum (Gibco, Waltham, MA, USA). Cell lines were cultured at 37 °C with 5% CO_2_. 3-deazaadenosine (DAA) (CAS No.1338466-77-5) was purchased from Sigma Chemical Co., (St. Louis, MO, USA).

### 2.3. Quantitative RT-PCR (qRT-PCR)

Total RNA was extracted from ovarian tissues and GCs using Trizol reagent (Invitrogen, Carlsbad, CA, USA). Reverse transcription was carried out in strict accordance with the manufacturer’s protocol. The specific procedure was as follows: Total RNA was extracted from GCs obtained from different groups strictly according to the instructions of the RNAiso Plus reagent kit (TaKaRa, Somerset, NJ, USA), and reverse transcription was subsequently carried out using the PrimeScript RT reagent Kit (TaKaRa, Dalian, China). The primers for qRT-PCR were synthesized by RiboBio (Guangzhou, China), and their sequences are listed in Table 1. *β-actin* was used as the endogenous control.

### 2.4. The Methylation Analysis by Sulfite Sequencing

The corresponding DNA samples were treated using the DNA bisulfite Conversion Kit (Tiangen Biochemical Technology Co., Ltd., Beijing, China). After treatment, the cytosine C at the CpG sites that did not undergo methylation and the C at the non-CpG sites in the DNA sample sequence were all converted to thymine T, while the C at the methylated CpG sites remained unchanged. The Methprimerer 1.0 (http://www.urogene.org/cgi-bin/methprimer/methprimer.cgi, accessed on 1 November 2025) software was used to search for regions rich in CpG sites near the *CYP11A1* gene promoter and convert the C at non-CpG sites into T. Subsequently, semi-nested PCR primers were designed on the transformed sequences, as follows: 5′-GGAAAGGAGTACGTCAAGGC (forward) and 3′-CGTACTGCAGCATGTGACTC (reverse). The target fragment amplified by the medial primers was purified and ligated with the pMD19-T vector (TaKaRa, Dalian, China) at 4 °C for 16 h. It was transferred to the competent cells of the top 10 Escherichia coli (BMED, Beijing, China), 500 µL of liquid medium was added, and it was cultured on a shaker at 37 °C and 170 r/min for 40 min. Subsequently, it was coated on ampicillin solid culture substrate plates containing isopropyl-β-d-thiogalactoside (IPTG) and X-gal and incubated at a constant temperature of 37 °C for 12 to 14 h. Positive monoclonal colonies were picked and placed in a liquid medium containing ampicillin and cultured at 37 °C and 170 r/min for 15 h. Colony PCR was performed on monoclonal strains using internal primers (IN-F and IN-R), with an annealing temperature of 51 °C. The PCR products were then processed and sequenced using the PyroMark Q48 ID Pyrosequencing system (Qiagen, Hilden, Germany). The amplification products were detected by electrophoresis using 1.5% agarose gel. At least 20 bacterial solutions with correct amplification bands were selected from each tissue and sent to Shanghai Sangon Biotech for sequencing. The sequencing results were viewed using Chromas 2.4.1 and DNAMAN 8.0. The methylation status of each CpG site in the amplified fragment was analyzed.

### 2.5. Cloning of Promoter Fragments and Vector Construction

Based on the *CYP11A1* promoter sequence obtained through cloning, the upstream primers of five truncated fragments were designed, respectively, at −1950 (PF1), −1552 (PF2), −1250 (PF3), −550 (PF4), and −233 (PF5) BPS using the software Primer Premier 6.0 (Table 1). Primer synthesis was carried out by Sangon Biotech (Shanghai, China) Co., Ltd. Downstream, the *CYP11A1* promoter amplification primer was used, and the *Kpn*I and *BgL*II (Abcm, Sydney NSW, Australia) digestion sites were added at the 5′ ends of the upstream and downstream primers, respectively. Using pMD19-T-*CYP11A1* (Promega, Madison, WI, USA) as the template, PCR amplification of truncated fragments of different lengths was carried out, and the amplification procedure was the same as that of the promoter clone part. The correctly sequenced truncated fragments were ligated with pMD19-T at 16 °C for 12 h. After transformation, monoclonal colonies were picked for expanded culture and plasmid extraction. The restriction digestion products were mixed with the pGL3-Basic vector (Invitrogen, Yingjie Company, Nanchang, China) and incubated at 4 °C to carry out an overnight ligation reaction. After the ligation product was transfected into target cells, the medium was replaced every 24 h during the subsequent cell culture process. The digestion products were ligated with the pGL3-Basic vector (Invitrogen) at 4 °C overnight. After transformation, the plasmids were extracted for enzyme digestion and identification, and the positive plasmids were handed over to Sangon Biotech (Shanghai, China) Co., Ltd. for sequencing.

### 2.6. Cell Transfection and Dual-Luciferase Activity Detection

GCs were cultivated in an incubator at 37 °C and 5% CO_2_. The medium was changed every 24 h. When the cell density reached 80% to 90%, they were transferred to 48-well plates. After 24 h of culture, the cell density reached 70–80%. Five recombinant plasmids with different deletion fragments were co-transfected with the renilla luciferase reporter gene vector pRLTK (Promega, Madison, USA) into sheep GCs (the mass ratio of recombinant plasmids to internal reference plasmids was 50:1). Cells were collected 48 h after transfection. Then, 20 µL of 1× PLB cell lysis buffer was added to each well. The cells were lysed at room temperature for 20 min. The luciferase activity was detected using the dual-luciferase reporter gene assay kit to determine the activity of each missing fragment.

### 2.7. Methylation Identification In Vitro

*M.Sss*I (NEB) can specifically recognize the CG site of the nucleotide sequence on double-stranded DNA, and S-adenosine methionine methylates the cytosine residue C. The experimental procedure was as described in a previous paper [18]. Briefly, the luciferase expression vector *CYP11A1*-211 of the *CYP11A1* gene was constructed by in vitro methylation modification. *M.Sss*I was added to the experimental group, while *M.Sss*I was not added to the control group, and water was added for supplementation. The methylation modification system is as follows: expression vector 2 µL, in vitro methylase *M.Sss*I 2 µL, S-adenosine methionine (SAM) 0.5 µL, 10× buffer 2 buffer solution 10 µL, ddH_2_O 85.5 µL, and the total system 100 µL. DNA was purified and recovered in a 37 °C water bath for 16 h, and the concentration was detected. Methylated and unmethylated plasmid DNA were treated with methylation-sensitive enzyme *Hpa*II (NEB).

### 2.8. Construction of CYP11A1 Overexpression Plasmid and siRNA

GCs were cultured in DMEM-F12 medium supplemented with 10% FBS at 37 °C in a 5% CO_2_ atmosphere. *CYP11A1* cDNA was cloned from human testis cDNA and inserted into the pcDNA3.1 plasmid with *Bam*HI and *Eco*RI restrictive enzyme sites. Lipofectamine3000 Transfection Reagent (L3000015, Invitrogen), following Invitrogen’s protocol, was used to transfect the GCs with pcDNA-3.1-*CYP11A1*. The transfection efficiency of pcDNA-3.1-*CYP11A1* into GCs was confirmed by Western blotting; an increasing amount of plasmid DNA (500 ng, 1250 ng, and 2000 ng) was transfected into BeWo cells to decide upon the optimal concentration. The role of *CYP11A1* expression during GCs was studied by transfecting GCs in vitro with the plasmid encoding using lipofectamine 3000 reagent (Invitrogen, Carsbad, CA, USA) or small interfering RNA (siRNA) against *CYP11A1* using lipofectamine RNAi MAX reagent (Invitrogen, Carsbad, USA) immediately after isolation. Sequences of *CYP11A1* siRNA were as follows: GCUG ACCAGUGACAAUGACTT (sense) and GUCAUUGUCA CUGGUCAGCTT (antisense) (GenePharma Co., Ltd., Suzhou, China). Randomly scrambled siRNA served as a negative control. GCs were collected for total RNA and protein extraction, and the conditioned cultured medium was collected for progesterone measurement 48 h after transfection.

### 2.9. Immunofluorescence

The expression intensity and pattern of *CYP11A1* in GCs were determined by immunofluorescence staining. After different treatments, cells, at RT, were fixed for 30 min with 4% (*w*/*v*) paraformaldehyde, permeabilized for 10 min with 0.1% Triton X-100 (T9284-500 ML, Sigma Life Science, Darmstadt, Germany), and then blocked with 1% (*w*/*v*) albumin bovine V (BS043E, Biosharp) for 1 h. The cells were incubated with the primary antibodies against *CYP11A1* (1:1000, Abcam, Cambridge, UK; catalog No. v175408, RRID: AB_2721042), followed by incubation with a goat anti-rabbit IgG secondary antibody (1 µg/mL, Alexa Fluor 488, A11008, Invitrogen) and goat anti-mouse fluorescence in isothiocyanateconjugated IgG (1:150, Invitrogen). The nuclei were labeled by DAPI (4′,6-diamidino-2-phenylindole1:1000). Cells were imaged by confocal microscopy (Olympus FV1000, Olympus, Tokyo, Japan). For immunostaining the mitochondria, an anti-Cox-IV antibody (Zen Bioscience, Chengdu, China) was used. Four random fields in three wells per group were counted (for the figures for the migrating cells) using Image J 2.14.0 software.

### 2.10. Western Blotting

Total cellular protein was extracted from cultured GCs using the radioimmunoprecipitation assay lysis buffer (Active Motif, Carlsbad, CA, USA) containing a mixture of phosphatase and protease inhibitors (Roche, Basel, Switzerland). After determination of protein concentration, the protein abundance of *CYP11A1* was determined following a standard protocol of Western blotting, as described previously [19].

Briefly, 30 μg of protein was electrophoresed in a 9% sodium dodecyl sulfate–polyacrylamide gel and transferred to the nitrocellulose membrane (Merck Millipore, Billerica, MA, USA). After blocking with 5% nonfat milk, the membrane was incubated with primary antibodies against *CYP11A1* (1:1000, Abcam; catalog No. v175408, RRID: AB_2721042) and *GAPDH* (1:10,000, Proteintech, Rosemont, IL, USA; catalog No. 60004-1, RRID: AB_2107436), respectively, overnight at 4 °C. After washing with Tween 20/ Tris-buffered salt solution, the membrane was incubated with horseradish peroxidase-conjugated corresponding secondary antibody (Proteintech) for 1 h. The bands with peroxidase activity were detected with a chemiluminescence detection system (Merck Millipore) and visualized using a G-Box chemiluminescence image capture system (Syngene, Bengaluru, Karnataka). The ratio of band density of *CYP11A1* to that of GAPDH was calculated to indicate the abundance of *CYP11A1*.

### 2.11. CCK-8 Assay

Cells were plated in 96-well plates at a density of 5 × 10^3^ cells in 100 µL medium per well 24 h before the experiment. The cell viability was examined by a CCK-8 kit (Dojindo Laboratories, Kumamoto, Japan) according to the manufacturer’s instruction.

### 2.12. Statistical Analysis

The SPSS19.0 software package and GraphPad Prism 7 (GraphPad Software, La Jolla, CA, USA) were used to perform all statistical analysis. Log-rank test was used for Kaplan–Meier survival analysis. Data are expressed as the mean ± SD of at least 3 independent experiments, and statistical evaluation was performed using one-way analysis of variance (ANOVA) or Student’s *t*-tests. Values of *p* < 0.05 or *p* < 0.01 are considered statistically significant.

### 2.13. Availability of Data and Materials

The datasets generated and/or analyzed during the current study are available from the corresponding author on reasonable request.

## 3. Results

### 3.1. Cellular Localization and Expression Analysis of the CYP11A1 Gene in GCs of Follicles with Different Diameters

**Figure 1:**
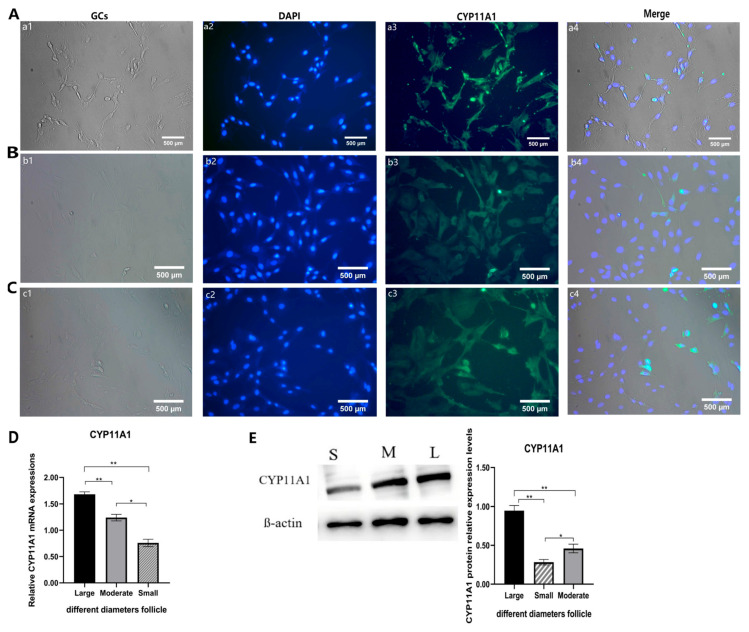
The localization and expression of the CYP11A1 in GCs of follicles with different diameters. Immunofluorescence results showing the expression and localization of CYP11A1 during GCs of follicles with different diameters (**A**–**C**). (**A**) The GCs in S for CYP11A1 (Control, **a1**), nuclei (DAPI, **a2**), anti-CYP11A1 (anti-CYP11A1, **a3**), and Merge (**a4**). (**B**) The GCs in M for CYP11A1 (Control, **b1**), nuclei (DAPI, **b2**), anti-CYP11A1 (anti-CYP11A1, **b3**), and Merge (**b4**). (**C**) The GCs in L for CYP11A1 (Control, **c1**), nuclei (DAPI, **c2**), anti-CYP11A1 (anti-CYP11A1, **c3**), and Merge (**c4**) Scale bars, 500 μm for all panels. QRT-PCR results showing the level changes of *CYP11A1* transcripts during the GCs in S, M, and L (**D**). WB results showing the level changes of CYP11A1 translation during the GCs in S, M, and L (**E**). Data are presented as mean ± SD. * *p* < 0.05; ** *p* < 0.01. n = 3 independent experiments (mean ± SD).

*CYP11A1* is the first key rate-limiting enzyme in steroid synthetic hormones and plays a crucial role in the growth and development of GCs. However, the role of CYP11A1 in GCs of follicles of different diameters remains unclear, as does the key window period of its role. Therefore, in this experiment, we divided follicles of different diameters into the following three groups: S (Small, 2~3.4 mm), M (Moderate3.5~5.4 mm), and L (Large follicles > 5.5 mm), and GCs of follicles of different diameters were isolated and obtained. Cellular immunofluorescence staining (IF) was used to locate CYP11A1 in follicular GCs of different diameters, as well as observation with an inversion microscope (10×). The results showed that the positive signals of CYP11A1 were distributed in the cytoplasm, nucleus, and mitochondria in GCs of Qira Black sheep (Figure 1A–C). The GCs in the S, M, and L groups were further detected by qRT-PCR and Western blot methods. The results (Figure 1D,E) revealed that the transcriptional and translation levels of *CYP11A1* in GCs in L were extremely significantly higher than those in the M and S groups (*p* < 0.01). The expression level of *CYP11A1* in M was significantly higher than that in group S (*p* < 0.05). These results suggest that the expression of *CYP11A1* may be related to the dominant selection of follicles, and the expression changes in this gene in GCs are related to the manipulation process of initiation. The follicular development period and physiological state are closely related to the reproductive state. GCs, as key follicular steroid secretory cells, through mitosis and continuous proliferation, participate in follicle growth, development, and maturity and a series of complex physiological processes. Further exploration of the function of the promoter region is needed to confirm this hypothesis.

### 3.2. Sequence Analysis of the 5′-Regulatory Region of the CYP11A1 Gene

**Figure 2:**
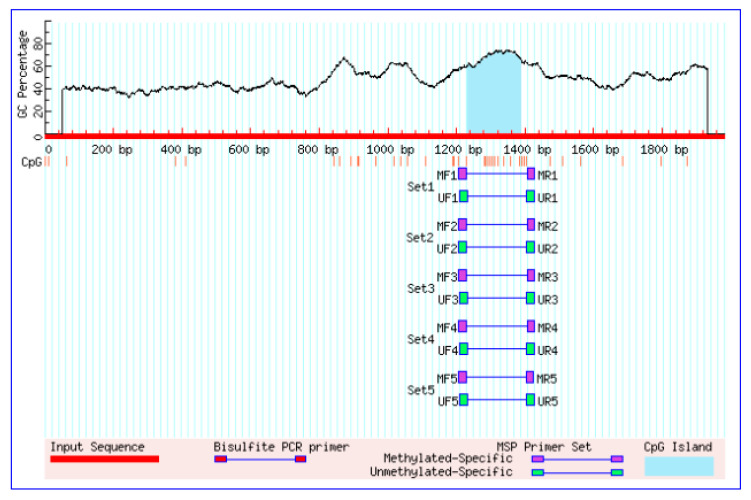
Prediction of promoter CpG island in the *CYP11A1* gene of ovisaries.

Studies have found that there are significant differences in the expression levels of *CYP11A1* among different developmental GCs, but the genetic mechanism of these differences remains unclear. It is speculated that the transcriptional regulation of the *CYP11A1* gene is affected by the methylation of its promoter. In order to further reveal the expression regulatory mechanism of *CYP11A1* in different developmental GCs, in this experiment, GCs from follicles of different diameters in Qira Black sheep were used as materials, and the methylation status of the *CYP11A1* gene promoter in GCs was detected by BSP technology, with the aim of exploring the expression regulatory mechanism of the *CYP11A1* gene during the development of GCs from the perspective of DNA methylation. Firstly, bioinformatics methods were used to search for the 5′-UTR nucleotide sequence of the *CYP11A1* gene in sheep from the UCSC database (http://genome.ucsc.edu/, accessed on 1 November 2025). Using on-line analysis software PromoterScan (Promoter 2.0—DTU Health Tech—Bioinformatic Services) and BDGP (http://www.fruitfly.org/seq_tools/promoter.html, accessed on 1 November 2025), the promoter region of the *CYP11A1* gene in sheep was predicted. The results showed that the promoter region of *CYP11A1* was located at −2060 to +1 nt (length 2060 bp, with the start codon AUG as +1). We used the online software MethPrimer 1.0 (http://www.urogene.org/cgi-bin/methprimer/methprimer_results.cgi, accessed on 1 November 2025) to separately predict the *CYP11A1* gene 5 ‘CpG island–control area. Among them, there was one CpG island in the 5′-regulatory region of the *CYP11A1* gene, with a length of 211 bp, respectively (Figure 2), indicating that the expression differences in the *CYP11A1* gene in GCs of follicles of different diameters may be related to the regulation of DNA methylation in its promoter region.

### 3.3. Methylation Analysis of the Promoter Region of the CYP11A1 Gene

**Figure 3:**
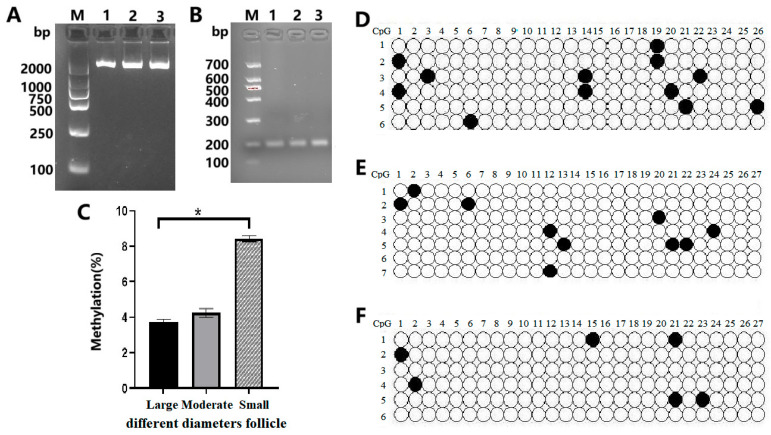
The *CYP11A1* gene promoter and its CpG island BSP amplification product in different-diameter follicular GCs of Qira Black sheep (**A**,**B**). Methylation-level expression of the *CYP11A1* gene in GCs of follicles with different diameters (**C**). Prompter methylation status the *CYP11A1* gene in GCs of the different-diameter follicles (**D**–**F**). (**A**) 1: Primers in the GCs of Large Follicles, 2: Primers in the GCs of Moderate Follicles, 3: Primers in the GCs of Small Follicles, M: Marker. (**B**) 1–3, respectively, represent the GCs of Large Follicles, Moderate Follicles, and Small Follicles. M Marker. (**C**) * indicates significant difference (*p* < 0.05). (**D**) Promoter methylation status of the *CYP11A1* gene in GCs in Large Follicles, (**E**) prompter methylation status of the *CYP11A1* gene in GCs in Moderate Follicles. (**F**) Promoter methylation status of the *CYP11A1* gene in GCs in Large Follicles. (**D**–**F**) The black circles represent the CG sites that have been methylated, while the white circles represent the CG sites that have not been methylated.

Based on the prediction of the promoter region and CpG island of the *CYP11A1* gene, primers were designed (Table 1). Using sodium bisulfite-modified genomic DNA from GCs of Qira black sheep follicles of different diameters as a template, the promoter region of the *CYP11A1* gene was amplified by PCR and sequenced (BSP). The AGAR gel electrophoresis detection results of the BSP amplification products are shown in Figure 3A,B. It can be seen from Figure 3A,B that the electrophoresis bands are clear and single, and the fragment size is consistent with expectations, allowing for subsequent operations. Our further analysis of the methylation molecules in the promoter region of the *CYP11A1* gene in GCs of three groups of follicles with different diameters (L, M, and S) is shown in Figure 3C. The total length of BSP amplification of the *CYP11A1* gene was 211bp, including two CpG sites. The methylation detection results showed that the methylation levels of the two CpG sites of the *CYP11A1* gene in GCs of L, M, and S were 3.70%, 4.23%, and 8.37%, respectively, all in a light methylation state (Figure 3D–F). This indicates that there are no differentially methylated regions in this area. However, with the increase in follicular diameter, the methylation degree of the *CYP11A1* promoter gradually increased. Among them, there was no significant difference between L and M (*p* > 0.05), while there was a significant difference between L and S (*p* < 0.05), and no significant difference between M and S (*p* > 0.05). It is speculated that the GCs’ imprinted expression of the *CYP11A1* gene in L, M, and S may be regulated by other epigenetic modifications such as methylation. This study enriched the number of *CYP11A1* genes in sheep and can provide a reference basis for further research on the function and imprinting regulatory mechanism of the *CYP11A1* gene.

### 3.4. Cloning and Enzyme Digestion Identification of the CYP11A1 Gene Promoter

**Figure 4:**
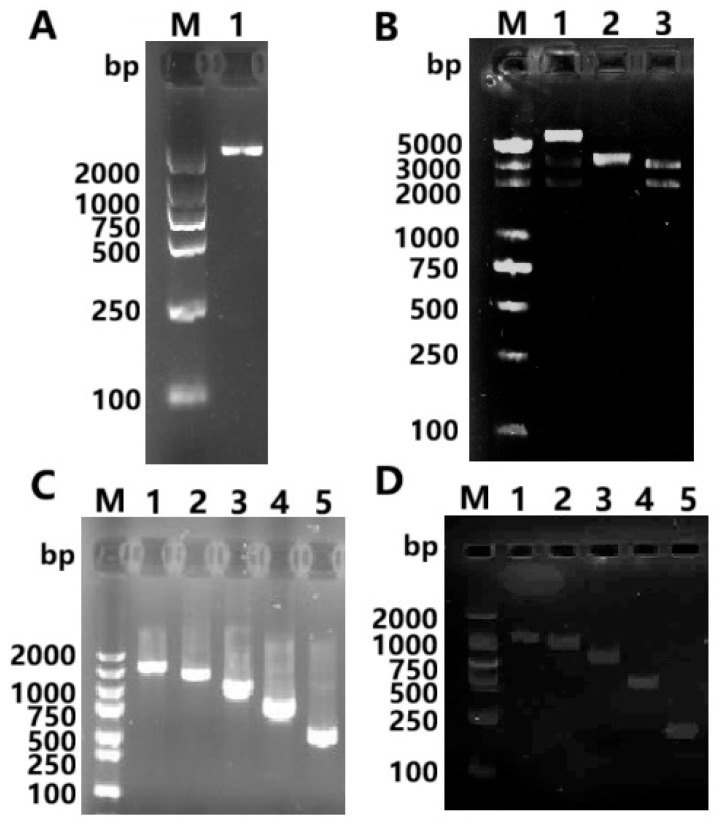
*CYP11A1* promoter restriction and fragmental amplification. Ovine *CYP11A1* gene promoter cloning (**A**) and enzyme digestion (**B**), M: DL5000DNAMarker; 1: pMD19T-*CYP11A1*; 2: pMD19T; 3: pMD19T-*CYP11A1* enzyme digestion product. (**C**) The amplification of different length fragments of *CYP11A1* promoter, (**D**) identification of the recombinant plasmids fragment by enzymes digestion. 1: pCYP11A1(−1905/+155); 2: pCYP11A1(−1552/+155); 3: pCYP11A1(−1250/+155); 4: pCYP11A1(−550/+155); 5: pCYP11A1(−233/+155).

PCR amplification was performed using the genomic DNA of sheep GCs as a template. The product was detected by 1% agarose gel electrophoresis, and a single band could be seen. The full length of the 5′ regulatory region of the *CYP11A1* gene promoter is 2060 bp (Figure 4A). The obtained *CYP11A1* promoter fragment was ligated with the pMD19-T vector to construct a clonal vector. Enzymatic digestion identification revealed the target fragment at 2060 bp (Figure 4B).

### 3.5. Methylation Analysis of the CYP11A1 Promoter Region in GCs

In order to further confirm the relationship between the DNA methylation status in the promoter region and the expression of the *CYP11A1* gene, the cloned *CYP11A1* promoter fragment was used as a template, and the genomic DNA of GCs in the sheep L group was utilized as a template. Primers (Table 1) were used to amplify the *CYP11A1* gene promoter region sequences of different lengths for the construction of the truncated expression vector. The agarose gel electrophoresis map of the PCR products is shown in Figure 4C. It can be seen from the figure that the target band is consistent with the expected size, and sequencing confirmed that the amplification result is correct. The promoter regions of the *CYP11A1* gene were of five different lengths (*CYP11A1* (−1905/+155), *CYP11A1* (−1552/+155), *CYP11A1* (−1250/+155), *CYP11A1* (−550/+155), *CYP11A1* (−233/+155)). They were ligated with the luciferase double reporter vector PGL-Basic after double enzymatic digestion with *Xho*I and *Hind*III, respectively, to construct the truncated expression vector of the *CYP11A1* gene promoter region in GCs. The result is shown in Figure 4D. It can be seen from the figure that the fragments obtained by enzymatic digestion are consistent with the expected length. The truncated expression vectors of the *CYP11A1* gene promoter in GCs were successfully constructed, namely *CYP11A1* (−1905/+155), *CYP11A1* (−1552/+155), *CYP11A1* (−1250/+155), *CYP11A1* (−550/+155), and *CYP11A1* (−233/+155).

### 3.6. The CYP11A1 Promoter Activity Was Detected by Dual-Luciferase

**Figure 5:**
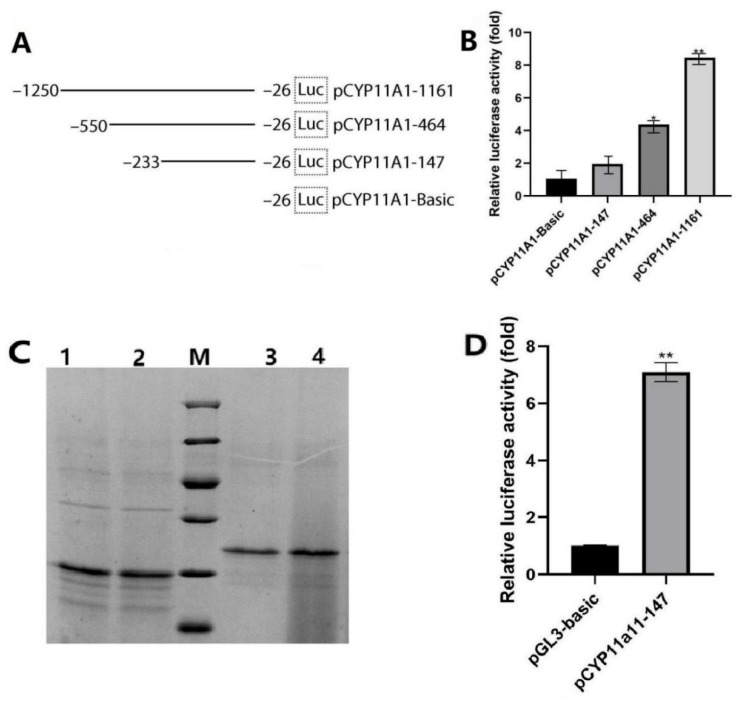
The CYP11A1 gene promoter activity analysis of granulosa cells in ovis aries and in vitro methylation affect core promoter region of the CYP11A1 gene. (**A**) Structure of expression vectors; (**B**) promoter activity; * indicates significant difference (*p* < 0.05); ** indicates extremely significant difference (*p* < 0.01). (**C**) Identification of recombinant vectors by restriction enzymes, AM: DL2000DNA marker; (**A**) 1–2: pCYP11A1 control group, 3–4: SssI methylation reaction performed on pCYP11A1-550. (**D**) The promoter region of the CYP11A1 gene was treated in GCs, and luciferase activity reflecting relative expression was measured 24 h after transfection with the expression vector. A histogram depicting the expression levels in GCs is presented, ** indicates extremely significant difference (*p* < 0.01). n = 3 independent experiments (mean ± SD).

Using pRL-TK as the internal reference plasmid, the recombinant five plasmids and the empty plasmid pGL3-basic were transfected into 293T and sheep GCs, respectively, using Lip3000. The truncated expression vector of the *CYP11A1* gene promoter in GCs underwent transient transfection into GCs. After 24 h, each component of the GCs was collected for luciferase activity analysis. The results showed (Figure 5A) that after transfection of 293T cells and the construction of five vectors starting from pCYP11A1-464 (−550/+155), the luciferase activity began to be significantly higher than that of the control group (*p* < 0.05), among which the luciferase activity of pCYP11A1-1540 (−1552/+60) was the highest (ZNF263). Sheep GCs were transfected and treated with DMSO at a concentration lower than 1% as a negative control. The results showed (Figure 5B) that starting from pCYP11A1-464 (−550/+155), the activity of the transfection group was significantly increased compared with the empty plasmid. It can be found from Figure 5A,B that there may be a positive regulatory element (HoXD10) in each of the −464/+155 regions of the 5′-regulatory region of the *CYP11A1* gene in GCs.

### 3.7. The Effect of In Vitro Methylation of the Core Promoter Region on the Activity of the CYP11A1 Gene Promoter Region in Sheep GCs

The core promoter expression vector of GCs’ *CYP11A1* gene (pCYP11A1-385) was treated with *M.Sss*I methyltransferase to methylate the CpG site. After purification of the product, the treatment effect of methyltransferase was identified by digestion with methyl-insensitive restriction endonuclease *Hpa*II. The results are shown in Figure 5C,D. As can be seen from Figure 5C, in the *M.Sss*I methyltransferase treatment group (lanes), the *Hpa*II cleavage site in the expression vector is protected, preventing the methylation-sensitive restriction endonuclease *Hpa*II from being cleaved. In contrast, the control group (lanes) is cleaved into many DNA fragments of different sizes, indicating that the treatment effect of *M.Sss*I methyltransferase is better. By taking an internal carrier, PRL–TK, there was a Margaret spellings *M.Sss*I methyltransferase core *CYP11A1* gene promoter region in the GCs’ treatment with the expression vector *CYP11A1* transient transfected into GCs (−550/+155). In addition, 24 h after testing relative expressed luciferase activity, with the results shown in Figure 5D, it was found that after methylation treatment, the core promoter activity of the *CYP11A1* gene promoter was extremely significantly decreased (*p* < 0.01).

### 3.8. The Effects of Overexpression with the CYP11A1 Gene on Proliferation in Sheep GCs

**Figure 6:**
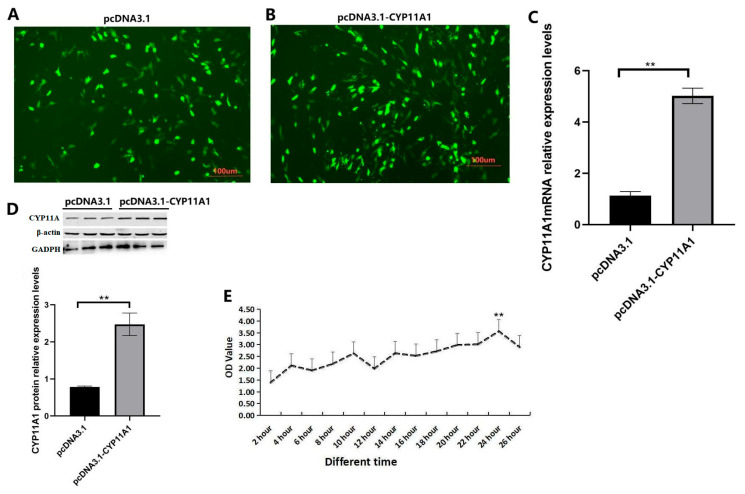
The effect of over-expression of the *CYP11A1* gene on GCs. (**A**) The transfected in GCs of negative pcDNA3.1 vector molecule (pcDNA3.1-*GFP*) was used as the control, (**B**) the pcDNA3.1-*CYP11A1* vector was further transfected into sheep GCs cultured in vitro. (**C**) The expression of *CYP11A1* gene in GCs of different components was detected by qRT-PCR, showing the histogram of mRNA abundance of *CYP11A1* gene over-expressed in follicular GCs. (**D**) The expression of CYP11A1 protein in GCs of different components was detected by WB, showing the gray scale graph of protein over-expression of CYP11A1, where the inner reference proteins are β-actin and GADPH. (**E**) The GCs’ proliferation level increased significantly at 24 h. ** indicates *p* < 0.01. n = 3 independent experiments (mean ± SD).

The PCDNA3.1-*CYP11A1* vector was further transfected into sheep GCs cultured in vitro. Additionally, the transfected negative pcDNA3.1 vector molecule (pcDNA3.1-*GFP*) was used as the control (Figure 6A,B). Then, 24 h after transfection, GCs in each group were collected, respectively. The expression of the *CYP11A1* gene in GCs of different components was detected by qRT-PCR and WB. The results are shown in Figure 6C,D. After overexpression of the *CYP11A1* gene, the relative expression levels of *CYP11A1* mRNA and protein in GCs showed multiple changes compared with the detection levels of the control group, which were significantly higher than those of the control group (*p* < 0.05). CCK-8 test showed that after the CYP11A1 expression, testing different time stages (2 h, 4 h, 6 h, 8 h, 10 h, 12 h, 14 h, 16 h, 18 h, 20 h, 22 h, and 24 h) of 26 GCs’ proliferation found that over time, GCs’ activity was on the rise. It reached the highest at 24 h and was significantly higher than that in other time periods (*p* < 0.05) (Figure 6E). The GCs’ proliferation level increased significantly at 24 h (Figure 6E).

### 3.9. The Effects of Interference with the CYP11A1 Gene on Proliferation in Sheep GCs

**Figure 7:**
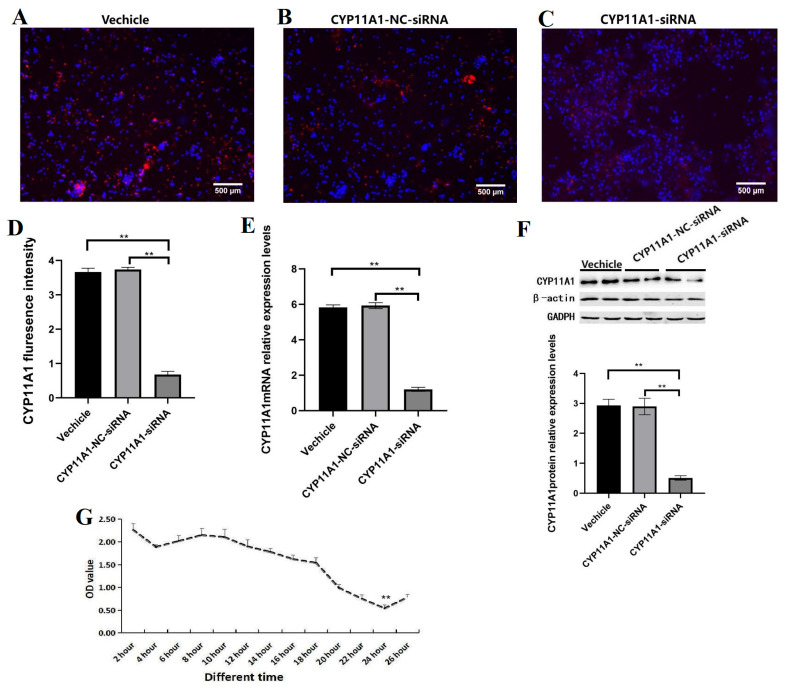
Effects of *CYP11A1*-siRNA gene interference on GCs. (**A**) GCs transfected with empty vector served as the control, (**B**) GCs transfected with *CYP11A1*-NC-siRNA) as negative control, (**C**) GCs transfected with *CYP11A1*-siRNA-211, (**D**) immunofluorescence assay detected the expression of CYP11A1 in GCs with different components, presenting a histogram of expression levels in GCs, (**E**) qRT-PCR detected the expression of *CYP11A1* in GCs with different components, showing a histogram of mRNA abundance after interfering with *CYP11A1*-siRNA gene in GCs, (**F**) WB detected the expression of CYP11A1 in GCs with different components, presenting grayscale images of protein over-expression of CYP11A1-siRNA gene. Reference proteins were β-actin and GAPDH. (**G**) After knocking out the CYP11A1 gene, the proliferation ability of GCs was detected at different time points (2 h, 4 h, 6 h, 8 h, 10 h, 12 h, 14 h, 16 h, 18 h, 20 h, 22 h, 24 h, 26 h). The proliferation level of GCs significantly decreased at 24 h. ** indicates *p* < 0.01. n = 3 independent experiments (mean ± SD).

Further determination was required to ascertain whether the change in the expression of the *CYP11A1* gene was directly related to the proliferation of GCs and whether it was the target gene in the proliferation process of GCs. Therefore, this experiment designed a specific siRNA sequence targeting the *CYP11A1* gene ((CYP11A1-sirNA-211:) Sense: GCAAGUGUAGAGAUAGUUACC Anti–Sense: UAACUAUCUCUACACUUGCGU). The solvent RNA enzyme-free water (Vehicle) and the negative siRNA molecule (*CYP11A1*-NC-siRNA) were transfected by cell transfection technology as controls (Figure 7A,B), and the *CYP11A1*-siRNA-211 molecule was transfected into GCs developed in vitro (Figure 7C). The protein level of CYP11A1 in GCs of different treatment groups was detected by IF. The results showed that, compared with the Control, 48 h after transfection with *CYP11A1*-sirNA-211, the fluorescence signal intensity of the CYP11A1 protein in GCs at 24 h was significantly lower than that in the Vehicle and *CYP11A1*-NC-siRNA groups (*p* < 0.05) (Figure 7C), while the nucleus and cytoplasm of GCs showed obvious stagnation. However, there was no significant difference between the Vehicle and *CYP11A1*-NC-siRNA (*p* > 0.05) (Figure 7D). This indicates that *CYP11A1*-siRNA-211 has a significant inhibitory effect on the development of GCs, which may be caused by the oxidation imbalance of GCs. The expression of the *CYP11A1* gene in GCs of different components was detected by qRT-PCR and WB. The results are shown in Figure 7E,F. After interference of the *CYP11A1* gene, the relative expression levels of *CYP11A1* mRNA and protein in GCs showed multiple changes compared with the detection levels of the control group, which was significantly decreased compared to that of the control group (*p* < 0.05). The oxidation imbalance of GCs leads to premature ovarian failure in sheep and shortens the reproductive years of ewes in actual production. This suggests that subsequent studies can focus on the molecular mechanism of GCs’ oxidation by the *CYP11A1* gene. Based on the above results, it is further confirmed that CYP11A1 plays a key regulatory role in the regulation of GCs in sheep. These studies provide reliable data support for the implementation of this project. After knockdown of *CYP11A1*, the proliferation ability of GCs at different time stages (2 h, 4 h, 6 h, 8 h, 10 h, 12 h, 14 h, 16 h, 18 h, 20 h, 22 h, 24 h, and 26 h) was detected. It was found that with an increase in time, the activity of GCs showed a significant downward trend and reached the lowest at 24 h, significantly lower than in other time periods (*p* < 0.05) (Figure 7G). The above results indicate that CYP11A1 can enhance the proliferation ability of ovine GCs and promote the development process of the ovaries.

## 4. Discussion

GCs are important functional cells of follicles. However, as the dominant follicle grows, the ability of GCs to produce estradiol weakens, affecting their development. The expression of CYP11A1 is the main reason for follicles acquiring the ability to produce estrogen [20]. CYP11A1 is a stable, hydrophilic, relatively conserved protein with antioxidant and steroid hormone synthesis functions. When the follicle reaches approximately 1mm and is located in the secondary follicle, CYP11A1 is detected, and the function of CYP11A1 mainly occurs in the dominant follicle [21]. With a continuous increase in CYP11A1 activity, it reaches a significant level in the GCs of the follicles before ovulation in the final stage of the follicular phase. CYP11A1 is also expressed in GCs from primary follicles to pre-ovulatory follicles. The expression of CYP11A1 ensures that the conversion of GCs to TCs to produce estradiol has high activity, ultimately fulfilling the requirements for the growth and development of dominant follicles. In the later stage of the follicular phase, GCs acquire the ability to produce P4. The main reason is that FSH induces LHR generation on GCs and promotes the luteinization of GCs [22]. CYP11A1 is a stable, hydrophilic, relatively conserved protein with antioxidant and steroid hormone synthesis functions. Once the egg is released, GCs transform into granulocytic luteal cells, which have the typical ultrastructure of GCs that produce CYP11A1. The cytoplasm contains abundant smooth endoplasmic reticulum, and the mitochondria have tubular cristae containing a large number of lipid droplets. Before ovulation and shortly after ovulation, GCs express a large amount of CYP11A1, which has the ability to produce P4 and E2 and maintains the stable development function of GCs [23]. CYP11A1 is mainly expressed in the corpus luteum and GCs of the ovary. The transcription factor controlling the expression of *CYP11A1* in these cells seems to be LHR1 located in the follicular membrane cells [24]. LHR1 activates the transcription of *CYP11A1* through the binding site SF1RE in the proximal (−38/−46) and upstream UCRS (−1603/−1617) regions. GCs specifically disrupt the *LHR1* gene, resulting in reduced expressions of Star and CYP11A1 and decreased progesterone synthesis. This proves the importance of LHR1 in the expression of *CYP11A1* in the ovary and steroid production [25]. CREB1 and GATA-4 are crucial for the *CYP11A1* transcription of GCs in luminal follicles. In the early stage of follicular development, CREB1 and GATA-4 synergistically regulate *CYP11A1* in a camp-dependent manner. After ovulation, FRA2 replaces CREB1 to regulate the expression of *CYP11A1* in luteal GCs [26].

In this study, the RT-PCR direct sequencing method based on SNP was used to analyze the imprinting status in GCs of different diameters of sheep follicles (L, M, and S). It was found that the expression of the *CYP11A1* in GCs in the L, M, and S groups was different, indicating that the imprinting of the *CYP11A1* gene was specific in follicular cells at different developmental stages. DNA methylation is an important epigenetic modification that plays a crucial role in regulating the selective expression of genes in mammals and maintaining genomic stability to ensure normal life processes such as growth and development of the organism (Mattei et al., 2022) [27]. During ovarian development, hypomethylation of promoter DNA is the key to inducing gene expression, while both a reduction in transcriptional activity and hypermethylation of DNA can lead to the inhibition of gene activation. CpG islands are often located near transcriptional regulatory regions, especially in the promoter regions of genes. By adding -CH3 to cytosine at CpG sites, they prevent RNA polymerase from recognizing transcription factors, thereby blocking the transcriptional regulation of GCs’ proliferation and differentiation by genes in ovarian GCs [28,29]. Hypermethylation of the CpG site in the *CYP11A1* promoter region of the mouse ovary (increasing by 17.16~64.28%) can interrupt steroid production. Changes in the methylation levels of imprinted genes *H19* and *Peg3* may also lead to ovarian dysfunction [30]. This will be the subject of continued exploration of the intergenerational or cross-generational genetic mechanism of *CYP11A1* methylation on ovarian function in domestic animals, and intervention measures will be proposed regarding whether ovarian function impairment can be reversed by changing the *CYP11A1* methylation status. The *CYP11A1* imprinted gene may be regulated by the DMR (Differentially methylated Region) in the promoter region, affecting its expression [31]. In sheep, in this study, the CpG island of the *CYP11A1* gene promoter was analyzed by nicosulfate sequencing. It was found that the *CYP11A1* gene showed light methylation in GCs in L, M, and S. The degree of methylation decreased with an increase in follicular diameter, and there were differences between the L and M groups, while there were no differences in other groups. It is indicated that the imprinting of the *CYP11A1* gene in dominant follicular GCs may be related to DNA methylation modification, but it cannot be ruled out that it is also regulated by other epigenetic modifications.

The imprinting of the *CYP11A1* gene in the growing and developing ovaries may be regulated by other epigenetic modifications besides DNA methylation. Studies in human placental trophoblast cells have confirmed that the methylation rate of CpG sites in the *CYP11A1* promoter is significantly reduced, and the expression of DNA methyltransferase (DNMT1) and enrichment in the *CYP11A1* promoter are in trophoblast synthylation. DNMT1 not only increases the methylation of CpG sites in the *CYP11A1* promoter, but also reduces the expression of *CYP11A1* and the production of progesterone. Similarly, it was found that there were multiple C/EBPα binding sites in the promoters of *CYP11A1* and DNMT1, and C/EBPα played a dual role in the regulation of *CYP11A1* during the synthesis process. C/EBPα not only directly drives the expression of *CYP11A1*, but also indirectly drives the reduction in methylation at the CpG site of the *CYP11A1* promoter by down-regulating DNMT1, resulting in an increase in progesterone production during the synthesis process [32].

Our research results have enriched the imprinting function of the *CYP11A1* gene in GCs during sheep developmental stages, which can provide a reference basis for further studies on the function and imprinting regulatory mechanism of the *CYP11A1* gene. Further, the imprinting of the *CYP11A1* gene promoter was carried out for cloning, and the full length of the 5′ regulatory region of the promoter was obtained, which was 2060bp. The truncated expression vectors of five promoter regions of different lengths of the *CYP11A1* gene were successfully constructed. It can be found from the research results that there may be a positive regulatory element (HoXD10) in each of the −464/+155 regions of the 5′-regulatory region of the *CYP11A1* gene in GCs. The relative expression activity of luciferase was detected 24 h later. After methylation treatment, the core promoter activity of the *CYP11A1* gene promoter was extremely significantly reduced. The *CYP11A1* gene in sheep GCs is controlled by another unique set of transcription factors. Most of the cis-regulatory elements and transcription factors of *CYP11A1* transcription were obtained through the study of promoter deletion and the analysis of DNA-binding proteins in electrophoretic transfer. Through precise regulation of CYP11A1 gene expression, it may be possible to improve reproductive traits in sheep, offering molecular targets for breeding new varieties with high reproductive performance. In molecular breeding applications, the regulatory element information of this gene can be used to develop genetic markers, facilitating the selection of breeding sheep with superior reproductive traits. Although these data are valuable, it should be noted that the shorter promoter fragments of *CYP11A1* were obtained from the endogenous promoter environment, so further evaluation is needed. We further conducted over-expression/RNAi on the shorter promoter fragment of *CYP11A1*. After 24 h of in vitro GC transfection, it was found that 24 h after overexpression of the *CYP11A1* gene, the relative expression levels of *CYP11A1* mRNA and protein in GCs showed multiple changes compared with the truncated levels of the control group, which were significantly higher than those of the control group. The cell proliferation level increased significantly at 24 h. Conversely, after 48 h of *CYP11A1*-siRNA-211, the fluorescence signal intensity of CYP11A1 protein in GCs was significantly lower than that in the Vehicle and *CYP11A1*-NC-siRNA groups. This indicates that *CYP11A1*-siRNA-211 has a significant inhibitory effect on the development of GCs. Researchers speculate that the most likely cause of this phenomenon may be the oxidative imbalance of GCs. The oxidation imbalance of GCs leads to premature ovarian failure in sheep and shortens the reproductive years of ewes in actual production. This suggests that subsequent studies can focus on the molecular mechanism of GCs’ oxidation by the *CYP11A1* gene. Based on the above results, it is further confirmed that CYP11A1 plays a key regulatory role in the regulation of GCs in sheep. These studies provide reliable data support for the implementation of this project.

## Figures and Tables

**Table 1:** Primers for transcription factor binding sites of *CYP11A1* in GCs of Qira Black sheep.

Gene Name	Primer Name	Primer Sequences (5′→3′)	Products Size (bp)	Annealing Tm/°C
Sheep *CYP11A1* (GenBank ID:100048994)	CYP11A1-388	F:GAAATGCTGGGCTGAATG R:TTGTCGGCAATGTCTCTG	233	58.0
	CYP11A1-705	F:ATGTGAAATGCTGGGCTG R:ATCTCGTCCTCTGTCATCCC	550	59.0
	CYP11A1-1405	F:ATGTGAAATGCTGGGCTG R:GTGTCTGTTGTTTGGGATTAGC	1250	59.0
	CYP11A1-1707	F:AGTGAAGAGGAACTAAAGAGCC R:AACATCAAGCATCAGCCAC	1552	58.0
	CYP11A1-2060	F:GGAAAGGAGTACGTCAAGGC R:CGTACTGCAGCATGTGACTC	1950	58.7

## Data Availability

The original contributions presented in this study are included in the article. Further inquiries can be directed to the corresponding author.
